# A proteomic analysis of chondrogenic, osteogenic and tenogenic constructs from ageing mesenchymal stem cells

**DOI:** 10.1186/s13287-016-0384-2

**Published:** 2016-09-14

**Authors:** Mandy J. Peffers, John Collins, John Loughlin, Carole Proctor, Peter D. Clegg

**Affiliations:** 1 Institute of Ageing and Chronic Disease, University of Liverpool, Leahurst, Chester High Road, Neston, CH64 7TE UK; 2 Musculoskeletal Research Group, Institute of Cellular Medicine, Newcastle University, Newcastle upon Tyne, NE2 4HH UK; 3 Newcastle University Institute for Ageing, Newcastle University, Newcastle upon Tyne, NE4 5PL UK; 4 Department of Musculoskeletal Biology, Institute of Ageing and Chronic Disease, The University of Liverpool, Leahurst, Neston, CH64 7TE UK

**Keywords:** Mesenchymal stem cells, Chondrogenesis, Osteogenesis, Tenogenesis, Ageing, Label-free quantification, Oxidative stress

## Abstract

**Background:**

Mesenchymal stem cells (MSCs) have prospective applications in regenerative medicine and tissue engineering but to what extent phenotype and differentiation capacity alter with ageing is uncertain. Consequently, any loss in functionality with age would have profound consequences for the maintenance of tissue viability and the quality of tissues. Proteomics enables the set of proteins responsible for a particular cell phenotype to be identified, as well as enabling insights into mechanisms responsible for age-related alterations in musculoskeletal tissues. Few proteomic studies have been undertaken regarding age-related effects on tissue engineered into cartilage and bone, and none for tendon. This study provides a proteome inventory for chondrogenic, osteogenic and tenogenic constructs synthesised from human MSCs, and elucidates proteomic alterations as a consequence of donor age.

**Methods:**

Human bone-marrow derived MSCs from young (*n* = 4, 21.8 years ± 2.4SD) and old (*n* = 4, 65.5 years ± 8.3SD) donors were used to make chondrogenic, osteogenic and tenogenic tissue-engineered constructs. We utilised an analytical method relying on extracted peptide intensities as a label-free approach for peptide quantitation by liquid chromatography–mass spectrometry. Results were validated using western blotting.

**Results:**

We identified proteins that were differentially expressed with ageing; 128 proteins in chondrogenic constructs, 207 in tenogenic constructs and four in osteogenic constructs. Differentially regulated proteins were subjected to bioinformatic analysis to ascertain their molecular functions and the signalling pathways. For all construct types, age-affected proteins were involved in altered cell survival and death, and antioxidant and cytoskeletal changes. Energy and protein metabolism were the principle pathways affected in tenogenic constructs, whereas lipid metabolism was strongly affected in chondrogenic constructs and mitochondrial dysfunction in osteogenic constructs.

**Conclusions:**

Our results imply that further work on MSC-based therapeutics for the older population needs to focus on oxidative stress protection. The differentially regulated proteome characterised by this study can potentially guide translational research specifically aimed at effective clinical interventions.

**Electronic supplementary material:**

The online version of this article (doi:10.1186/s13287-016-0384-2) contains supplementary material, which is available to authorized users.

## Background

Mesenchymal stem cells (MSCs) are a heterogeneous population of multipotent cells with the ability to differentiate into cells of mesenchymal origin to accommodate tissue homeostasis and repair. These cells have prospective applications in clinical tissue regenerative strategies, due to their potential to differentiate into musculoskeletal cell lineages, including cartilage, bone and tendon [1, 2], high ex-vivo expansion capacity and relative ease of culture following isolation [3]. The principles of tissue engineering involve a complex interplay of factors and to what extent MSC phenotype and differentiation capacity alter with ageing is unknown. Consequently, any loss in functionality with age would have profound consequences for the maintenance of tissue viability and the quality of tissues. The capacity of MSCs to differentiate into various types of tissue seems to change with age [4, 5]. For example, MSCs in older age are impaired with respect to osteogenic differentiation, preferring the adipogenic pathway of differentiation [6]. However, many current research findings are conflicting. For instance, relating to chondrogenic differentiation of MSCs, one study identified a reduction in glycosaminoglycan with age [7] whereas another found no change [5]. Furthermore, in osteogenic differentiation one group identified an increase in alkaline phosphatase with age [8] whilst another demonstrated a decrease [9]. It is thought these discrepancies could be due to the heterogeneous population which is propagated within and amongst donor populations. One recent study demonstrated that commercially isolated MSCs had a shorter doubling time than freshly isolated cultures. This may be due to commercially isolated cells having a more homogeneous population, eliminating uncertainty that heterogeneous freshly isolated populations enact on MSC characterisation [10].

The regenerative capacity of MSCs is believed to be due to their high proliferation and differentiation capacities, paracrine action (such as the secretion of plasminogen activators and matrix metalloproteases; important for extracellular matrix (ECM) remodelling), and immunological privilege. However, therapeutic effectiveness of MSCs for most clinical applications remains limited, possibly due to the attenuation of their regenerative potential in aged patients with chronic diseases. Nevertheless, MSCs have already been used in clinical trials of cell therapy for cartilage repair and osteoarthritis (reviewed in [11]), bone fracture healing [12] and in a limited number of tendon therapies [13, 14].

Because of the interest in MSCs for regenerative medicine, and with an ever ageing population it is important to understand how the sets of proteins in MSC-derived musculoskeletal ‘tissue’ change with age, and what the potential effects are on clinical outcome. In a recent review, the basic protein inventory of a characteristic MSC has been assembled which encompasses six functional groups of proteins [15]. Further work has identified the protein changes in osteogenic [16], myogenic [17], chondrogenic [18] and adipogenic differentiation [19] from bone-marrow-derived MSCs (BM-MSCs).

Proteomics enables the set of proteins responsible for a particular cell phenotype to be identified, as well as enabling insights into mechanisms responsible for age-related alterations in musculoskeletal tissues [20]. Label-free quantification proteomics methods are based on the direct measurement of the mass spectrum (mass to charge ratio of ions in the gas phase) acquired signal. When constituent peptides are produced following protein digestion and are converted into ions, the most abundant proteins will produce the most ions and thus the greatest signal intensities [21]. This type of workflow coupled with a high mass resolution mass spectrometer and bioinformatics enables hundreds of proteins to be identified and quantified in a single sample with high specificity and sensitivity [22].

Little information is available concerning the cellular and ECM protein changes that occur when BM-MSCs from different biologically aged donors are differentiated into chondrogenic or osteogenic constructs and no data are available for tenogenic constructs. This is important because tissue engineering aims to develop biomimetic tissues that recapitulate biological, structural and functional characteristics of native tissue. Age-related changes thus have potential implications for the tissue-engineering strategies used for enhancing musculoskeletal repair. This study aims to evaluate and compare the proteome of chondrogenic, osteogenic and tenogenic constructs derived from young and old human BM-MSCs in order to determine similar and distinct changes in the construct proteome with ageing.

## Methods

All chemicals are supplied by Sigma unless stated otherwise. Human MSCs from young and old donors were purchased from Stem Cell Technologies (Grenoble, France) and Promocell (Heidelberg, Germany).

### Cell culture and differentiation

Human bone-marrow-derived commercially available MSCs from young (*n* = 4, 21.8 years ± 2.4SD) and old (*n* = 4, 65.5 years ± 8.3SD) donors were grown to passage 4, and each donor was differentiated into chondrogenic, osteogenic [16] and tenogenic [23] constructs as described previously and used in all subsequent experiments [24]. Engineered tendon constructs were formed in six-well plates coated with ~1.5 ml SYLGARD (WPI, Hertfordshire, UK) and pinned with staples. For each construct, 0.4 ml of 0.6 × 10^6^ cells/ml was suspended in 80 μl of 20 mg/ml fibrinogen to which 8 μl of 200 U/ml thrombin was added. Then 160 μl of mixture was placed in each well to form an even covering. Each cell-embedded fibrin gel was cultured in 2 ml DMEM supplemented with 100 units/ml penicillin/streptomycin, 10 % FBS, 500 ng/ml amphotericin, 2 mM l-glutamine, 200 μM l-ascorbic acid 2-phosphate, non-essential amino acid at 10 μl/ml concentration and aprotinin at 10 μl/ml. Every other day the edges of the constructs were scored using a fine pipette tip and the media were changed. All constructs were fully contracted between the staples and harvested at 28 days post seeding [23]. All tissue culture was undertaken in 5 % oxygen and media were changed every third day. MSCs from each donor were analysed separately in the three lineages and proteomic analysis was carried out on all preparations for each of eight donors.

### Validation of differentiation

Differentiation was assessed for chrondrogenesis and osteogenesis by comparison with control MSCs treated identically except with maintenance media (complete Dulbecco’s Modified Eagle’s Media; Gibco) using histology and quantitative real-time PCR (qRT-PCR). Calcium depositions, a marker of bone formation and differentiation, were determined in osteogenic constructs using Alizarin red staining as described previously [25]. Chondrogenic pellets were paraffin embedded and 4 μm sections taken and further stained with Alcian Blue/Nuclear Fast Red. Tendon constructs were fixed in 4 % paraformaldehyde, longitudinally embedded in paraffin and 4 μm sectioned on polylysine slides. Staining was undertaken with Masson’s Trichrome (collagen) [26].

Transmission electron microscopy (TEM) of tendon constructs was performed by fixation in 2.5 % glutaraldehyde in 0.1 M sodium cacodylate buffer for 8 hours, followed by buffer washing procedure and second fixation and contrast stain with 0.1 % osmium tetroxide for 90 minutes. Samples were stained with 8 % uranyl acetate in 0.69 % maleic acid for 90 minutes, dehydrated in ascending ethanol concentrations and embedded in epoxy resin. Ultrathin cross-sections (60–90 nm) were cut with a Reichert-Jung Ultracut on an ultramicrotome using a diamond knife. Cut cross-sections were then mounted on 200 mesh copper grids and stained with ‘Reynold’s Lead citrate’ stain for 4 minutes. Images were viewed in Philips EM208S Transmission Electron Microscope at 80 k.

Total RNA was prepared from constructs using 0.5 ml Tri Reagent (Ambion, Warrington, UK) per construct with homogenisation using a fine needle and syringe. The guanidinium thiocyanate–phenol–chloroform extraction technique was used as described previously [27]. M-MLV reverse transcriptase and random hexamer oligonucleotides were used to synthesise cDNA from RNA (Promega, Southampton, UK) in a 25 ml reaction. Aliquots of 1 ml were amplified by PCR in 20 ml reaction volumes on an ABI 7300 Sequence Detector using a SYBR Green PCR mastermix (Applied Biosystems, Warrington, UK). cDNA was used for lineage-specific gene expression markers using qRT-PCR relative to *GAPDH* [28]. Steady-state transcript abundance of potential endogenous control genes was measured in the RNAseq data (unpublished data). *GAPDH* was selected as the most stable endogenous control gene. Primers used are presented in Additional file 1: Table S1.

### Protein extraction and sample preparation

Proteins were extracted from the constructs using either guanidine hydrochloride as described previously [20] (chondrogenic and osteogenic) or 0.1 % Rapigest™ [29] (tenogenic), following optimisation of protein extraction methods for each construct type (data not shown). Protein extracts were normalised following protein assay using the Bradford assay with Coomassie Plus™ protein assay reagent (Thermo Scientific, Rockford, IL, USA) read at 660 nm. In-solution trypsin digestion was undertaken on all samples as described previously [20]. Samples were desalted using C18 tips (Merck Millipore, Watford, UK) [30].

### One-dimensional SDS-PAGE

Construct soluble extracts of MSCs and constructs were analysed by one-dimensional sodium dodecyl sulfate polyacrylamide gel electrophoresis (SDS-PAGE) to assess gross quantitative/qualitative differences in protein profiles [20]. Additionally, Rapigest™ extracts of mesenchymal stems cells prior to differentiation were also evaluated. Then 30 μg was loaded according to equal volumes after ethanol precipitation and resolubilisation in SDS loading buffer (Invitrogen) and stained with Coomassie.

### Mass spectrometry and label-free quantification

Liquid chromatography tandem mass spectrometry (LC-MS/MS) was performed using a NanoAcquity™ ultraperformance LC (Waters, Manchester, UK) online to an LTQ-Orbitrap Velos mass spectrometer (Thermo-Fisher Scientific, Hemel Hempstead) as described previously [21]. The proteomics data were deposited to the ProteomeXchange Consortium [31] via the PRIDE partner repository with the dataset identifier PXD001952.

For label-free quantification the Thermo raw files of the acquired spectra from in-solution tryptic digests were analysed by the ProgenesisQI™ software (Version 1; Waters, Manchester, UK) [21]. Briefly, the top five spectra for each feature were exported from ProgenesisQI™ and utilised for peptide identification in PEAKS® 7 PTM (Bioinformatics Solutions Inc., Ontario, Canada) using the reviewed Uniprot human database. Search parameters used were: 10 ppm peptide mass tolerance and 0.6 Da fragment mass tolerance; one missed cleavage allowed; fixed modification, carbamidomethylation; and variable modifications, methionine, proline, lysine oxidation. Proteins were identified with a false discovery rate (FDR) of 1 % and a minimum of two peptides per protein. The resulting peptide-spectrum matches were imported into ProgenesisQI™ for label-free relative quantification. Differentially expressed (DE) proteins were defined with FDR *p* < 0.05 and ±2-fold regulation.

### Neopeptide identification

For neopeptide determination of tenogenic constructs, mass spectrometry data from the in-solution tryptic digests were analysed. Neopeptides were identified by searches against the reviewed Uniprot human database using Mascot with search parameters as already stated except: enzyme, semi-tryptic. Neopeptides were only included if they passed a conservatively applied threshold with *e* > 0.001. Patterns of fragmentation were determined for collagens, proteoglycans and glycoproteins.

### Functional analysis of proteomics data

To determine gene ontology, functional analyses, networks, canonical pathways and protein–protein interactions of age-related DE proteins in each construct type we performed the analyses using the Protein ANalysis THrough Evolutionary Relationships (PANTHER) Classification System [32], the functional analysis and clustering tool from the Database for Annotation, Visualisation, and Integrated Discovery (DAVID bioinformatics resources 6.7) [33] and Ingenuity Pathway Analysis (IPA) [34]. Proteins were further characterised and classified using MatrisomeDB [35].

### Western blotting validation of mass spectrometry data

To confirm the mass spectrometry data, the relative abundance of cartilage oligomeric matrix protein (COMP) and biglycan in tenogenic and superoxide dismutase 1 (SOD1) in the chrondrogenic protein extracts was determined using automated western blotting (Wes Simple Western Analysis; ProteinSimple, San Jose, CA, USA) as described previously [36]. All reagents were supplied by ProteinSimple. Simple western analysis was performed according to the user manual. Protocols were optimised for antibody and protein loading. In brief, protein extracts were mixed with a master mix to give a final concentration of 0.004–0.2 mg/ml total protein, 1× sample buffer, 1× fluorescent molecular weight markers and 40 mM dithiothreitol. Samples were heated at 95 °C for 5 min. Samples, blocking solution, primary antibodies, horseradish peroxidase-conjugated secondary antibodies, chemiluminescent substrate, and separation and stacking matrices were loaded into designated wells in a microplate. After plate loading, fully automated electrophoresis and immunodetection took place with the capillary system. Proteins were separated by molecular weight at 375 V for 25 minutes, and primary and secondary antibodies incubated for 30 minutes. All antibodies were diluted in antibody diluent to the required concentrations (Additional file 2: Table S2). Chemiluminescence was captured by a charge-coupled device camera, and the digital image was analysed using ProteinSimple Compass software. The relative amount of each protein, relative to total protein content, was calculated based on the peak area. A 29 kDa system control antibody was spiked into each sample to provide within-capillary normalisation. Statistical significance was determined using a Mann–Whitney test.

### Statistical analysis

Statistical analysis was undertaken with Mann–Whitney *U* tests for qRT-PCR and neopeptide analysis using GraphPad Prism version 6.0 (GraphPad Software, San Diego, CA, USA).

## Results

### Characterisation of tissue constructs

**Fig. 1:**
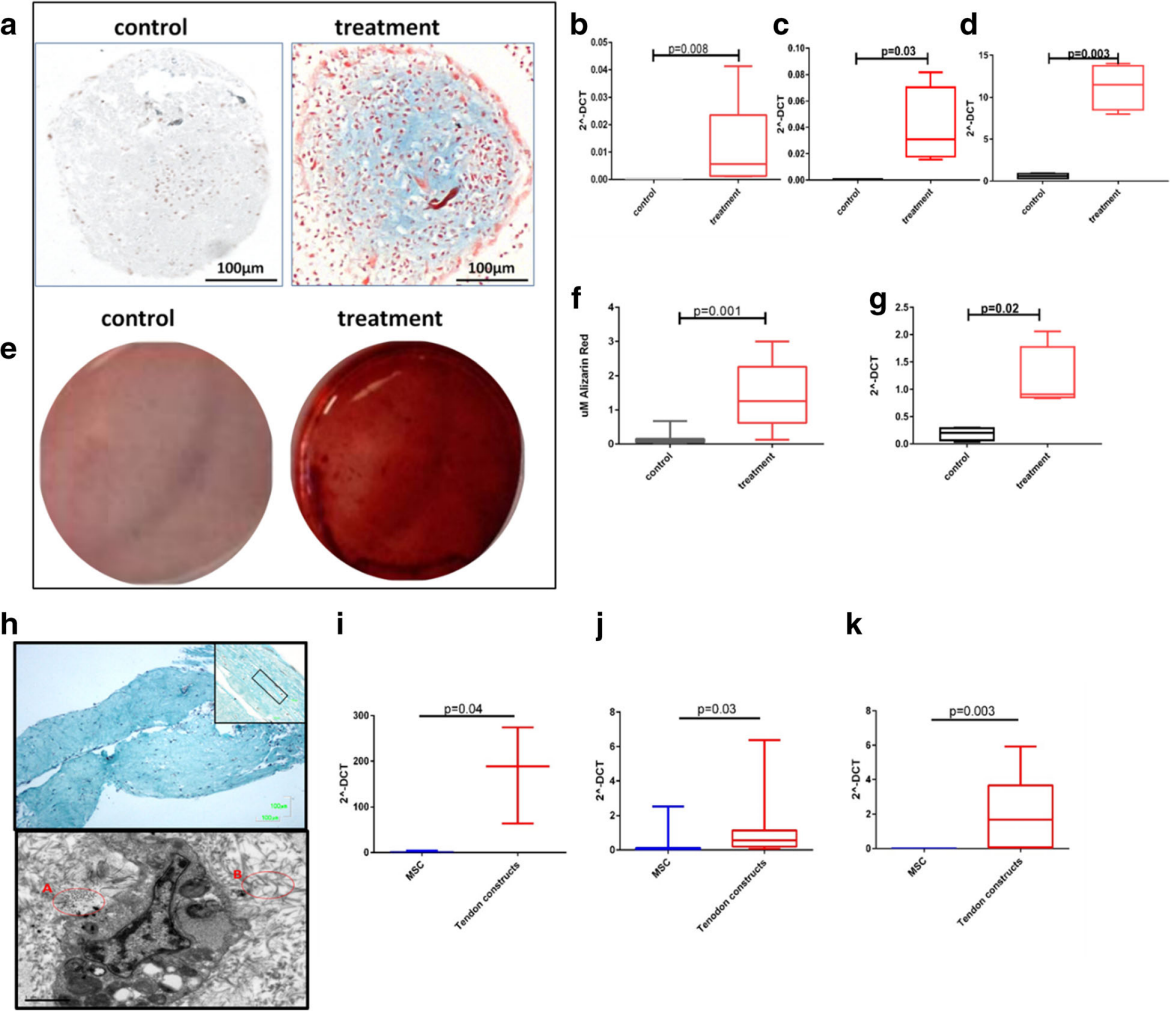
Histochemical and gene expression analysis of chondrogenic, osteogenic and tenogenic lineage differentiation for MSCs. Images are representative of all experiments. **a** MSC pellets cultured in control or chondrogenic media were fixed and stained with Alcian Blue (*scale bar* = 100 μm). Gene expression of **b** aggrecan, **c** *COL2A1* and **d** *SOX9* following chondrogenic differentiation. Statistical evaluation was undertaken using Mann–Whitney *U* test (*n* = 6). **e** Osteogenic differentiation from MSCs was confirmed with Alizarin Red S staining at day 21 to visualise mineralised bone matrix following extraction of the calcified mineral from the stained monolayer at low pH. **f** Box and whisker plot showing quantitative results of Alizarin red staining. Statistical significance, Mann–Whitney *U* test *p* < 0.001 (*n* = 12). **g** Gene expression of *RUNX2* following osteogenesis. **h** Histology images of a tendon construct made from MSCs stained with Masson’s Trichrome to identify collagenous matrix. Image was captured at ×4 magnification and ×10 magnification (*inset*, *upper image*) (*scale bar* = 100 μm). Example of more organised areas of collagen is marked on the inset image (*red*). (*Lower image*) Ultrastructural analysis using scanning TEM. Presence of aligned extracellular collagen fibrils (*A*) and less organised collagen (*B*) are inset (*red*) (*scale bar* = 1 μm). Tenogenic differentiation was also evaluated using gene expression of **i** *COL1A1*, **j** *SERPINF1* and **k** *THBS4*. For gene expression, data are represented as 2^–ΔCT^ compared with *GAPDH*. Statistical evaluation was undertaken using Mann–Whitney *U* test (*n* = 8) with data represented as 2^–ΔCT^ compared with *GAPDH. MSC* mesenchymal stem cell

To confirm chondrogenic induction of MSCs, mature markers of chondrocytes were assessed; Alcian Blue staining for glycosaminoglycans and aggrecan, *COL2A1* and *SOX9* gene expression. In line with previous reports [18] we identified an increase in Alcian Blue staining and aggrecan, *COL2A1* and *SOX9* expression [37] (Fig. 1a–d), demonstrating chondrogenic differentiation of MSCs. Osteogenic differentiation was evaluated with Alizarin Red and *RUNX2* gene expression. There was a significant increase in staining with Alizarin Red both visually and using quantitative analysis (Fig. 1e, f) and increased *RUNX2* expression (Fig. 1g), demonstrating osteogenic differentiation of MSCs. Tenogenic differentiation was evaluated histologically following Masson’s Trichrome staining, indicating areas of organised and disorganised collagen fibril formation within the constructs. This was confirmed with TEM and with gene expression of *COL1A1* (Fig. 1 h, i). There was no qualitative difference in the collagen organisation of tendon constructs derived from young and old MSCs. Further genes previously identified as markers of tendon expression [38] were significantly increased following tenogenic differentiation but were not age related; serpin peptidase inhibitor F (*SERPINF1*) (Fig. 1j) and thrombospondin 4 (*THBS4*) (Fig. 1k).

### SDS-PAGE comparative analysis of protein extracts

**Fig. 2:**
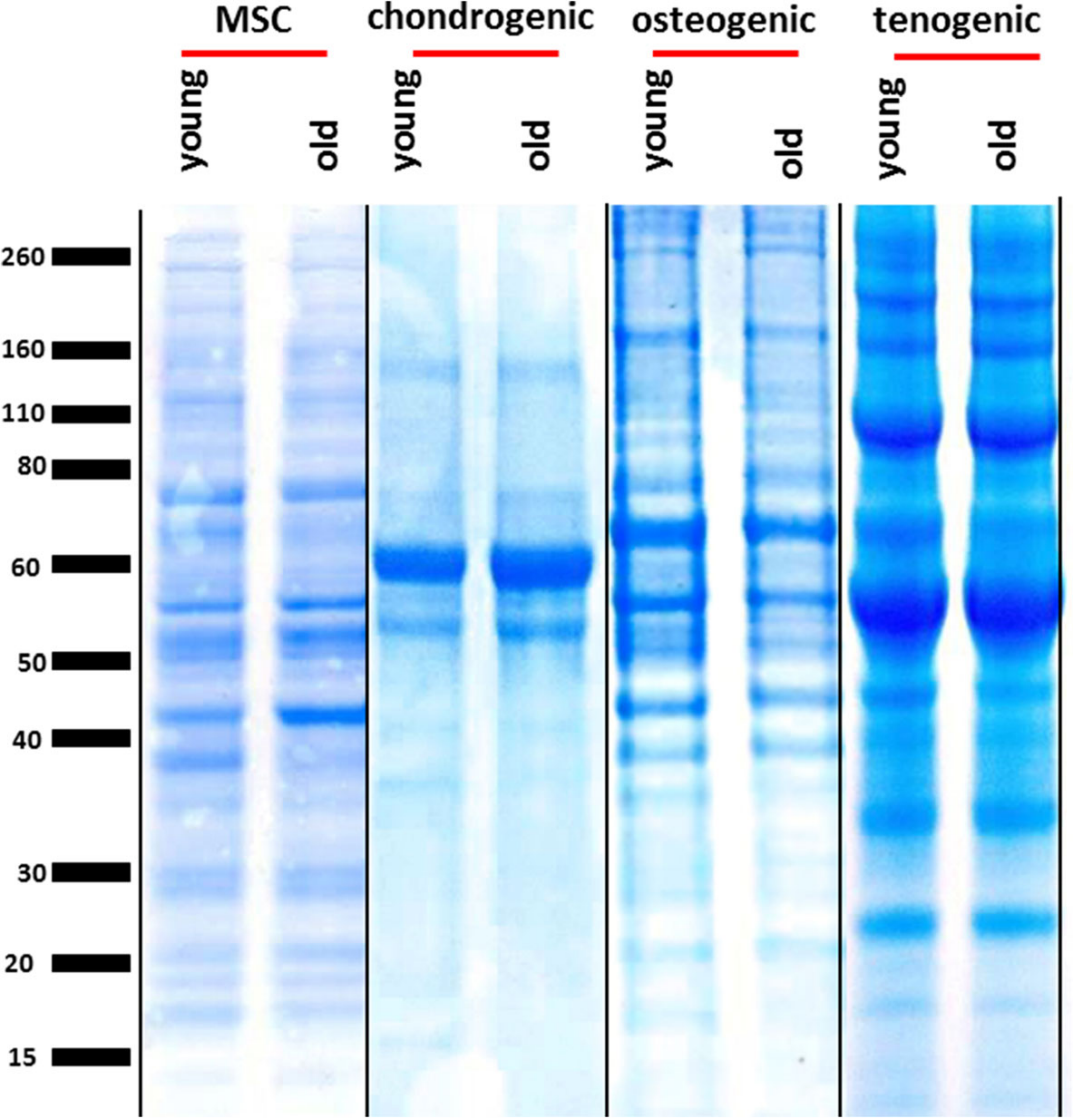
Coomassie-stained one-dimensional SDS-PAGE of the guanidine-soluble protein extracts of chrondrogenic and osteogenic constructs and Rapigest™ extracts of tenogenic constructs compared with Rapigest™ extract of MSCs. Images are representative of all experiments. Equal protein loading by weight (30 μg per well) allowed a qualitative and grossly quantitative comparison of soluble protein extracts. *Vertical black line* indicates independent gels. *Lines* indicate digital splicing*. MSC* mesenchymal stem cell

One-dimensional SDS-PAGE of the soluble protein extracts demonstrated differences in the intensity of the staining and the number of bands between construct type and MSCs but not with age. Osteogenic profiles were most similar to undifferentiated MSCs, showing more cellular profiles. The chondrogenic profiles were the least complex, as demonstrated by few stained bands (Fig. 2).

### Protein identification

A total of 2226 proteins for chondrogenic constructs, 2233 proteins for osteogenic constructs and 615 proteins for tenogenic constructs were identified within each construct type. Additional file 3: Table S3 presents detailed information on the identification of proteins in each. Each dataset was subjected to gene ontology using PANTHER which identified few differences in terms of gene ontological terms between each construct type (data not shown). Proteins in the ECM fraction were classified and further investigated using MatrisomeDB [35] (Additional file 3: Table S3). The overlap of proteins shared between construct types is shown Additional file 4: Figure S1. The percentage of matrisomal proteins compared with all proteins was 4 % for chondrogenic and osteogenic constructs and 11 % for tenogenic constructs.

### Label-free relative quantification

To compare relative protein levels between young and old chondrogenic, osteogenic and tenogenic constructs, samples were processed for LC-MS/MS, and label-free quantitative analysis undertaken using ProgenesisQI™. Each young and old sample from each lineage was analysed separately and then comparisons were made between all young and old donors for each lineage. Principal component analysis of all of the proteins identified revealed that the proteins clustered according to the age of the donor with a principal component for chondrogenic constructs of 71 %, osteogenic constructs of 66 % and tenogenic constructs of 83 %. The number of DE proteins with at least a 2-fold change, *p* < 0.05 and *q* < 0.05 was 128 in chondrogenic constructs (28 higher in old, 100 lower), 207 in tenogenic constructs (201 higher in old, six lower) and four in osteogenic constructs (three higher in old – gamma-adducin (ADDG), uveal autoantigen with coiled-coil domains and ankyrin repeats (HOYNH8), and heterogeneous nuclear ribonucleoprotein (ROA0) – one lower – aldehyde dehydrogenase X(ALB1)) (Additional file 5: Table S4).

### Gene ontology of DE proteins

**Fig. 3:**
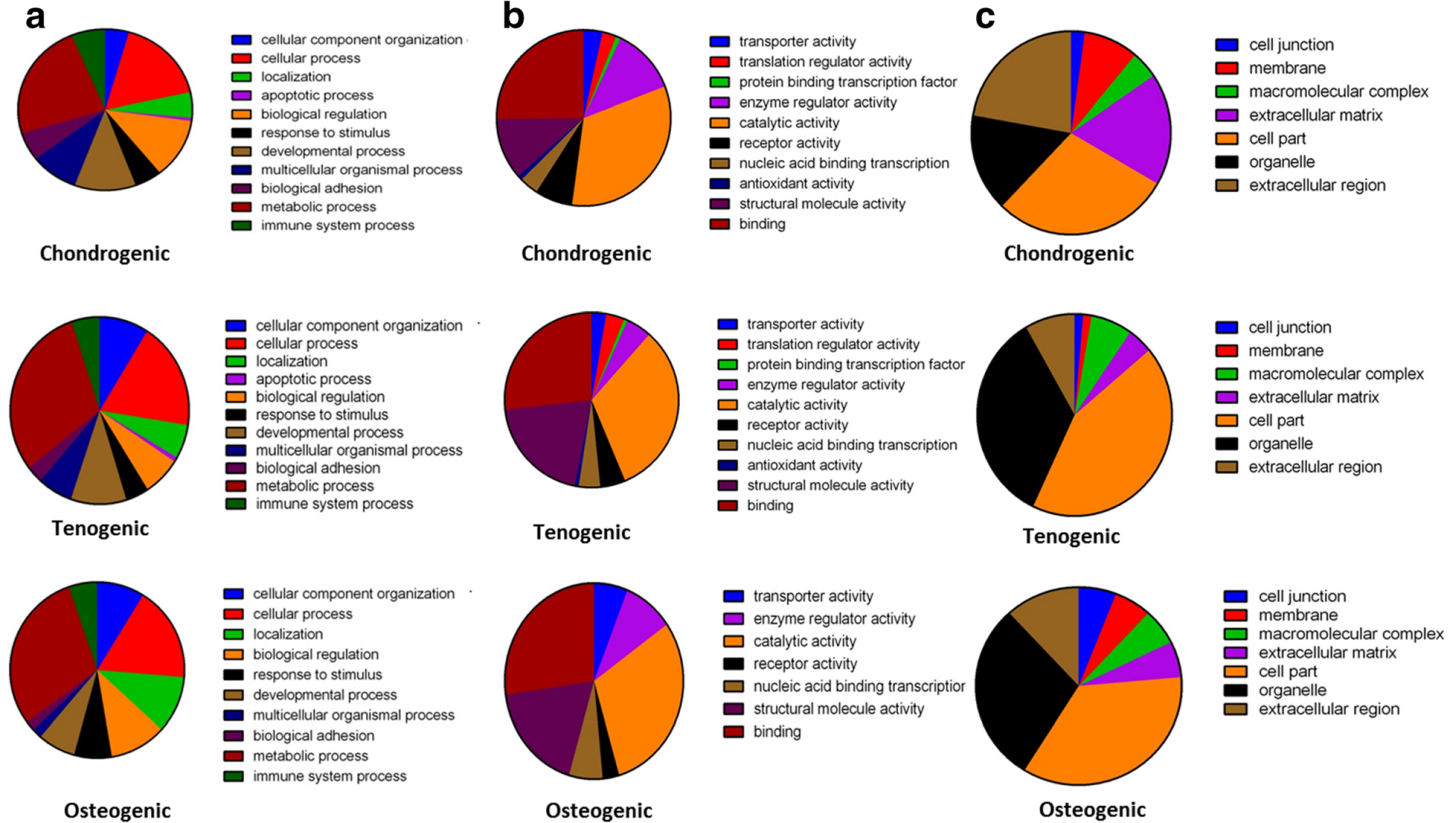
Pie charts depicting protein classification of DE proteins in ageing constructs using PANTHER. Proteins were demonstrated as DE when quantified with ProgenesisQI™ with at least two unique peptides, a 2-fold change in expression and *q* < 0.05. *First row*, chondrogenic constructs; *second row*, tenogenic constructs. **a** Biological processes, **b** molecular functions and **c** cellular components. Because osteogenic constructs had only four DE proteins with the filters of a 2-fold change in expression. *p* < 0.05 and *q* < 0.05, PANTHER analysis was undertaken on DE proteins quantified with ProgenesisQI™ with at least two unique peptides, a 2-fold change in expression and *p* < 0.05 (*third row*)

Using DAVID analysis we identified ontology terms which were shared and distinct between construct types. For gene ontology analysis of the DE proteins for chondrogenic and tenogenic constructs we used the parameters 2-fold change, *p* < 0.05 and *q* < 0.05. For osteogenic analysis, because only four proteins were DE at 2-fold change with *p* < 0.05 and *q* < 0.05, we investigated gene ontology terms and undertook IPA analysis for the 63 DE proteins with 2-fold change and *p* < 0.05 (Additional file 6: Table S5). A significant term common to all constructs was actin cytoskeleton organisation. For chondrogenic constructs DAVID terms were also principally involved in tissue morphogenesis and cell adhesion, and for tenogenic constructs these were principally glycolysis and protein metabolism. For osteogenic constructs the single significant term ‘ribonucleoproteins’ was identified (Additional file 7: Table S6). A similar number of DE matrisomal proteins was identified in chondrogenic and tenogenic constructs but none in osteogenic constructs (Additional file 3: Table S3). Four matrisomal proteins were DE in both chondrogenic and tenogenic constructs with age; COL4A2, matrix metalloproteinase 14 (MMP14), matrix remodelling associated 5 (MXRA5) and thrombospondin 1(THBS1). In osteogenic constructs, four matrisomal proteins were DE: WNT1 inducible signalling pathway protein 2 (WISP2), collagen type XV alpha 1 (COL15A1), proline/arginine-rich end leucine-rich repeat protein (PRELP) and serpin peptidase inhibitor (SERPINE2) (Additional file 3: Table S3). Proteins DE for chondrogenic and tenogenic constructs (2-fold change, *p* < 0.05 and *q* < 0.05) and osteogenic constructs (2-fold change and *p* < 0.05) were further classified for gene ontology with PANTHER, demonstrating both similarities and differences in the type of proteins affected with age (Fig. 3).

### Differential expressed genes and network analysis

**Fig. 4:**
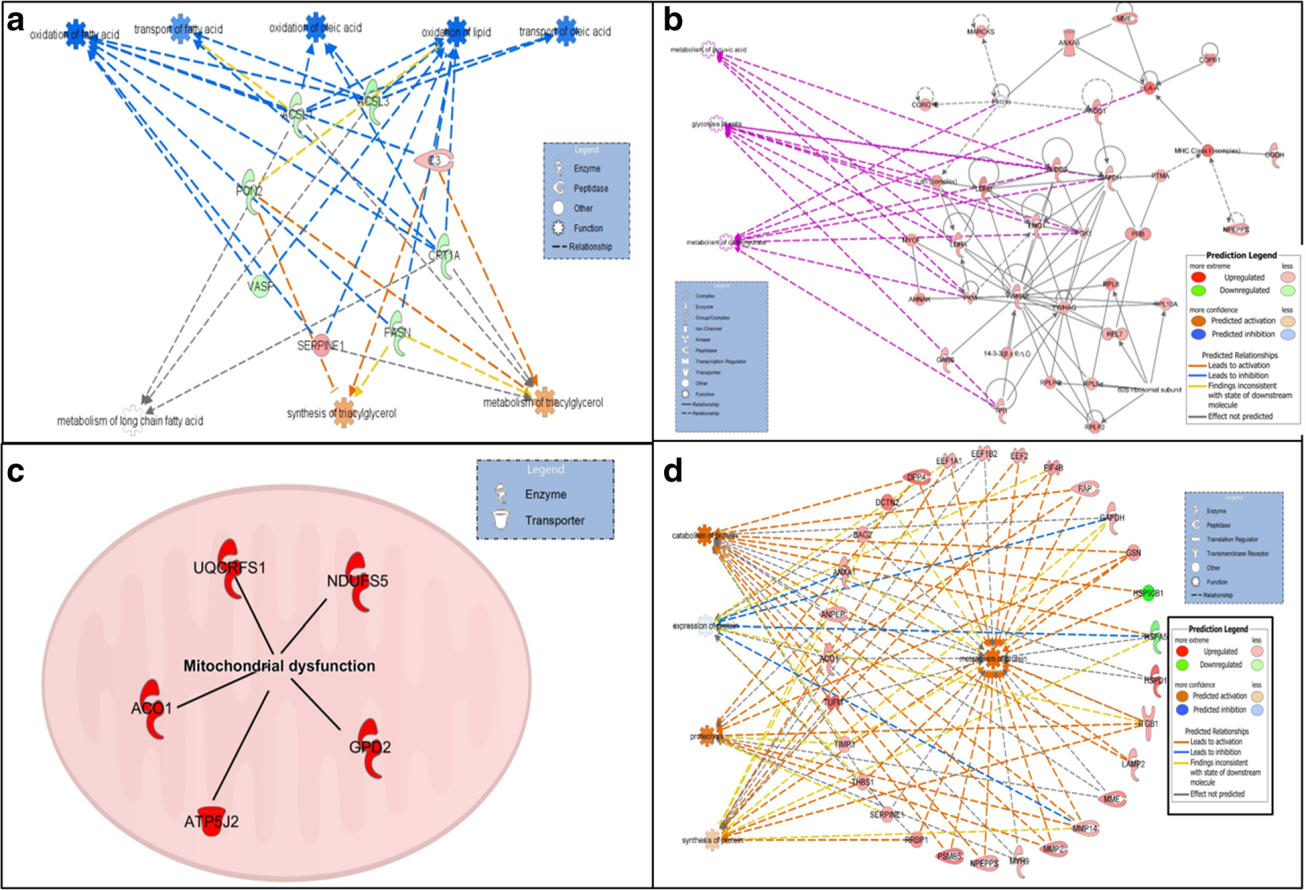
IPA generated networks derived from the proteins with different abundance in the chondrogenic, tenogenic and osteogenic constructs derived from young and old MSCs. IPA identified that lipid metabolism signalling pathways were predominant in chondrogenic constructs (**a**). In tenogenic constructs, signalling pathways were enriched for glucose metabolic processes (**b**). In osteogenic constructs, the principle signalling pathway was mitochondrial dysfunction (**c**). One of the principle functions associated with the DE proteins in tenogenic constructs was also protein metabolism (**d**). *Green nodes*, greater protein abundance in young; *red nodes*, greater protein abundance in old; *white nodes*, proteins not differentially abundant between young and old. *Intensity* of colour is related to higher fold-change. Key to the main features in the networks is shown

For chondrogenic and tenogenic constructs the sets of DE proteins associated with ageing were analysed with IPA. The top canonical pathways, networks and diseases and biological functions with ageing are presented in Tables 1, 2 and 3 respectively. From the DE proteins associated with ageing input into IPA, a number of potential upstream regulators were identified in chondrogenic, tenogenic and osteogenic constructs (Additional file 8: Table S7). The activation *z* score is used to infer likely activation states of upstream regulators based on comparison with a model that assigns random regulation directions. Only upstream regulators with significant activation *z* scores were investigated to increase confidence in the data. Interesting upstream regulatory analysis revealed that targets of SMAD-2, SMAD-3, SMAD-4 and transforming growth factor beta (TGFβ) are regulated in chondrogenic constructs. Here TGFβ was predicted to be inhibited with ageing, with effects on tissue development and chondrogenic differentiation. In contrast, TGFβ was identified in tenogenic constructs as an activating upstream regulator in ageing. Targets of hypoxia-induced factor-1 alpha (HIF1α) were activated or inhibited in the same way as TGFβ in chondrogenic and tenogenic constructs. Targets of epidermal growth factor receptor family (ERBB4) were differentially regulated in osteogenic constructs (Additional file 8: Table S7). Interestingly, age-related changes in signalling pathways for chondrogenic constructs were principally in lipid metabolism (Fig. 4a), for tenogenic constructs were in glucose metabolism (Fig. 4b) and for osteogenic constructs were in mitochondrial dysfunction (Fig. 4c). Furthermore, age-related alterations in functions in tenogenic constructs included protein metabolism (Fig. 4d). Table 4 summarises the functional categories, canonical pathways and networks identified from the changes in protein expression in each construct with ageing using IPA.

**Table 1:** Top canonical pathways from the IPA knowledge base that involve proteins differentially expressed in young compared with old chondrogenic, tenogenic and osteogenic constructs

Construct	Pathway	*p* value	Ratio
Chondrogenic^a^	LXR/RXR activation	6.01 × 10^–7^	8/121 (0.066)
	FXR/RXR activation	8.69 × 10^–7^	8/127 (0.062)
	Acute phase response signalling	7.36 × 10^–6^	8/169 (0.047)
	Cholesterol biosynthesis I	5.49 × 10^–5^	3/13 (0.231)
	Cholesterol biosynthesis II	5.49 × 10^–5^	3/13 (0.231)
Tenogenic^a^	Glycolysis I	5.15 × 10^–9^	7/25 (0.28)
	Gluconeogenesis I	1.85 × 10^–7^	6/25 (0.24)
	ILK signalling	6.45 × 10^–7^	12/186 (0.065)
	Mitochondrial dysfunction	1.96 × 10^–6^	11/171 (0.064)
	Protein ubiquitination pathway	3.10 × 10^–6^	13/255 (0.051)
Osteogenic^b^	Mitochondrial dysfunction	1.29 × 10^–4^	5/171 (0.029)
	Lipid antigen presentation by CD1	2.51 × 10^–3^	2/26 (0.077)
	Integrin signalling	2.68 × 10^–3^	4/202 (0.02)
	Oxidative phosphorylation	3.79 × 10^–3^	3/109 (0.028)
	Glycerol-3-phosphate shuttle	8.58 × 10^–3^	1/3 (0.333)

^a^Significant DE proteins with *p* < 0.05, *q* < 0.05 and ±2-fold change

^b^Significant DE proteins with *p* < 0.05 and ±2-fold change

*DE* differentially expressed, *IPA* ingenuity pathway analysis

**Table 2:** Top scoring networks from the IPA knowledge base that involve proteins differentially expressed in young compared with old chondrogenic, tenogenic and osteogenic constructs

Construct	Identification of associated network functions	Score
Chondrogenic^a^	Cell death and survival, lipid metabolism, small molecule biochemistry	43
	Cellular movement, cell death and survival, cancer	33
	Connective tissue disorders, haematological disease, hereditary disorder	31
	Cellular assembly and organisation, cellular development, connective tissue development and function	28
	Cell signalling, nucleic acid metabolism, small molecule biochemistry	26
Tenogenic^a^	Carbohydrate metabolism, haematological disease, immunological disease	59
	Developmental disorder, hereditary disorder, inflammatory disease	43
	Cell-to-cell signalling and interaction, embryonic development, tissue development	36
	Endocrine system development and function, energy production, small molecule biochemistry	36
	Cellular assembly and organisation, cellular function and maintenance, cellular compromise	31
Osteogenic^b^	Cellular assembly and organisation, tissue development, infectious disease	58
	Cellular compromise, developmental disorder, haematological disease	32
	Protein synthesis, developmental disorder, hereditary disorder	29
	Lipid metabolism, nucleic acid metabolism, small molecule biochemistry	8

^a^Significant DE proteins with *p* < 0.05, *q* < 0.05 and ±2-fold change

^b^Significant DE proteins with *p* < 0.05 and ±2-fold change

*DE* differentially expressed, *IPA* ingenuity pathway analysis

**Table 3:** Top diseases and biological functions from the IPA knowledge base that involve proteins differentially expressed in young compared with old chondrogenic, tenogenic and osteogenic constructs classified as diseases and disorders, molecular and cellular functions and physiological system development and function

Construct		p-value	No. molecules
Diseases and disorders			
Chondrogenic^a^	Inflammatory Response	1.14E-03 - 1.05E-08	34
	Developmental Disorder	7.11E-04 - 5.32E-08	33
	Skeletal and Muscular Disorders	5.85E-04 - 5.32E-08	46
	Cancer	1.21E-03 - 1.37E-07	112
	Organismal Injury and Abnormalities	1.21E-03 - 1.37E-07	113
Tenogenic^a^	Cancer	1.26E-18 - 1.84E-03	173
	Organismal Injury and Abnormalities	1.26E-18 - 1.96E-03	136
	Reproductive System Disease	1.26E-18 - 1.84E-03	104
	Immunological Disease	2.13E-12 - 5.18E-04	61
	Respiratory Disease	2.78E-12 - 3.26E-04	48
Osteogenic^b^	Infectious Disease	2.00E-05 - 1.54E-02	19
	Developmental Disorder	4.83E-05 - 1.14E-02	8
	Hereditary Disorder	4.83E-05 - 1.25E-02	11
	Metabolic Disease	4.83E-05 - 1.14E-02	10
	Neurological Disease	4.83E-05 - 1.77E-02	18
Molecular and cellular functions			
Chondrogenic^a^	Cell Death and Survival	1.05E-03 - 2.51E-15	67
	Cell Morphology	1.21E-03 - 7.47E-14	50
	Cellular Movement	1.24E-03 - 8.51E-12	52
	Cell-To-Cell Signalling and Interaction	1.21E-03 - 8.55E-12	45
	Cellular Assembly and Organization	1.24E-03 - 8.63E-12	49
Tenogenic^a^	Cellular Movement	3.79E-20 - 2.14E-03	85
	Cellular Growth and Proliferation	1.50E-18 - 1.91E-03	109
	Cell Death and Survival	1.91E-15 - 2.04E-03	98
	Cellular Assembly and Organization	1.62E-14 - 2.20E-03	85
	Cellular Function and Maintenance	1.62E-14 - 2.20E-03	87
Osteogenic^b^	Cell Morphology	2.33E-06 - 1.71E-02	19
	Lipid Metabolism	2.42E-05 - 1.71E-02	6
	Small Molecule Biochemistry	2.42E-05 - 1.72E-02	17
	Molecular Transport	2.94E-05 - 1.71E-02	22
	Protein Trafficking	2.94E-05 - 2.94E-02	5
Physiological system development and function			
Chondrogenic^a^	Connective Tissue Development and Function	1.21E-03 - 2.75E-10	45
	Tissue Development	1.24E-03 - 3.83E-09	60
	Organismal Survival	1.18E-04 - 7.09E-09	47
	Haematological System Development and Function	1.24E-03 - 1.05E-08	29
	Immune Cell Trafficking	1.24E-03 - 1.05E-08	27
Tenogenic^a^	Tissue Development	1.98E-08 - 2.22E-03	79
	Haematological System Development and Function	1.25E-07 - 1.47E-03	35
	Immune Cell Trafficking	1.25E-07 - 1.47E-03	32
	Cardiovascular System Development and Function	2.08E-07 - 2.11E-03	30
	Skeletal and Muscular System Development and Function	3.87E-07 - 2.11E-03	47
Osteogenic^b^	Nervous System Development and Function	2.33E-06 - 1.80E-02	12
	Organ Morphology	7.75E-05 - 1.71E-02	15
	Reproductive System Development and Function	7.75E-05 - 1.71E-02	7
	Tissue Development	1.15E-04 - 1.71E-02	16
	Cardiovascular System Development and Function	2.05E-04 - 1.59E-02	14

^a^Significant DE proteins with p < 0.05, q < 0.05 and ±2-fold change

^b^Significant DE proteins with p < 0.05 and ±2-fold change

*DE* differentially expressed, IPA ingenuity pathway analysis

**Table 4:** Summary of significant functional categories, canonical pathways and networks identified from DE proteins by IPA in constructs made from young or old MSCs for chondrogenic, tenogenic and osteogenic constructs

Biological processes	Chondrogenic^a^	Tenogenic^a^	Osteogenic^b^
Antioxidant changes	✓	✓	✓
Cell death and survival	✓	✓	✓
Cytoskeleton changes	✓	✓	✓
Energy metabolism	x	✓	✓
Protein metabolism	x	✓	✓
Lipid metabolism	✓	x	✓
Musculoskeletal abnormalities increased	✓	✓	x
Cell movement	✓	✓	✓
Cell proliferation	✓	✓	✓
Integrin signalling	x	✓	✓

^a^Significant DE proteins with *p* < 0.05, *q* < 0.05 and ±2-fold change

^b^Significant DE proteins with *p* < 0.05 and ±2-fold change

*DE* differentially expressed, *IPA* ingenuity pathway analysis, *MSC* mesenchymal stem cell

### Validation of mass spectrometry results by automated western blotting

**Fig. 5:**
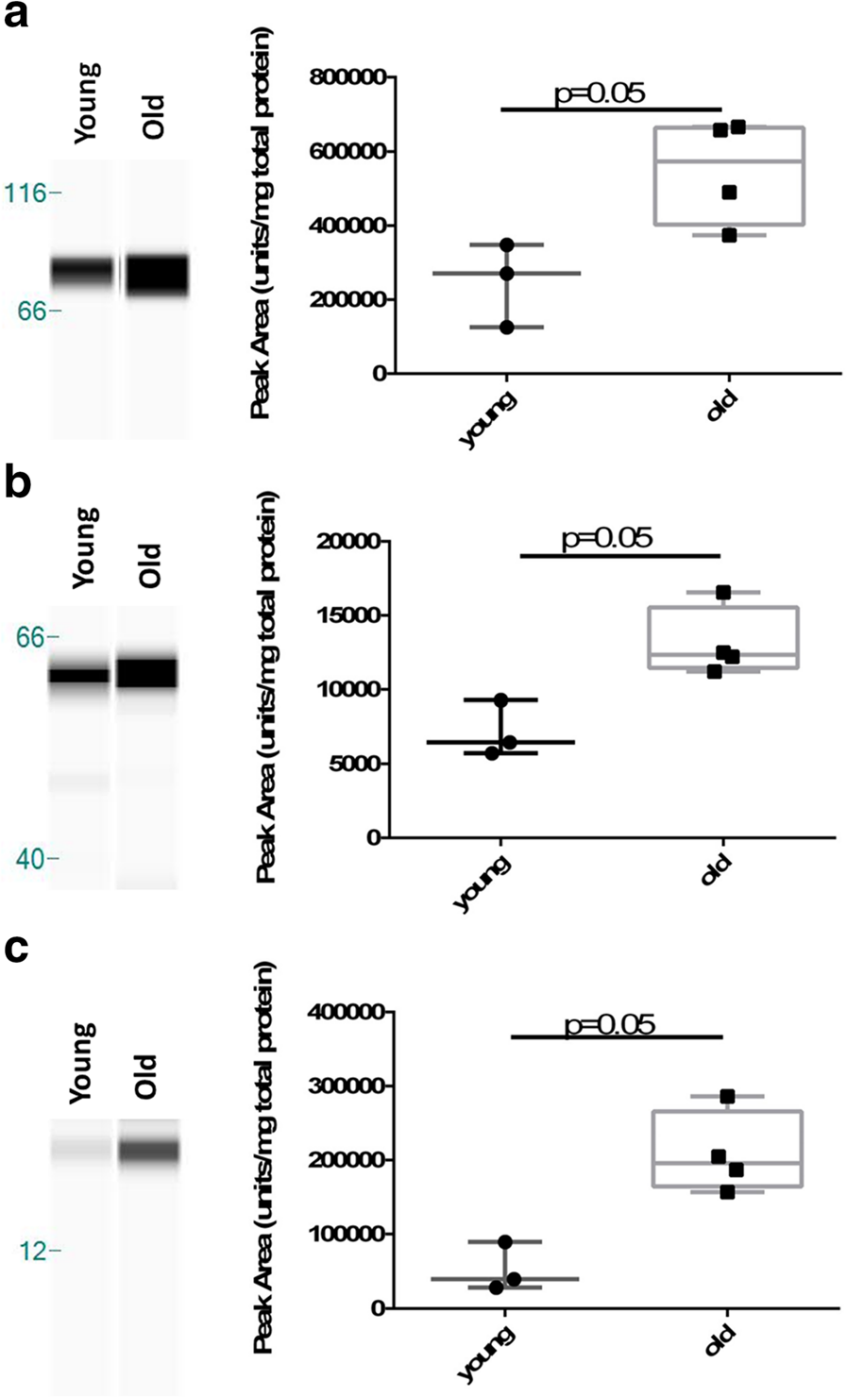
Western blotting validations of mass spectrometry results. COMP, biglycan and SOD1 abundance were confirmed by western blotting. Representative western blots for tenogenic constructs (**a** COMP and **c** biglycan) and chondrogenic constructs (**e** SOD1). Abundance of each protein is expressed semi-quantitatively (**b**, **d**, **f**) relative to total protein content. Statistical differences were assessed with age in the respective construct and antibody analysis using Mann–Whitney tests. Significant differences represented with *p* ≤ 0.05

Abundance of cartilage oligomeric matrix protein (COMP) and biglycan (tenogenic) and superoxide dismutase 1 (SOD1) was validated by western blotting (Fig. 5). Apparent molecular weights were larger than expected for COMP and biglycan. This is probably due to samples not being deglycosylated prior to western blotting, but this may also be due to evident differences in molecular weight (particularly for glycosylated proteins) using the ProteinSimple system for the majority of antibodies due to a difference in the separation matrix [39]. In agreement with the mass spectrometry data, for tenogenic constructs COMP and biglycan were higher in abundance (*p* = 0.05) in old constructs. SOD1 was higher in abundance (*p* = 0.05) in the old chondrogenic constructs, also supporting the mass spectrometry results.

### Identification of ECM fragmentation patterns in tendon constructs

Neopeptides were identified in all samples. Significantly more neopeptides were identified in the tendon constructs derived from old MSCs than from young MSCs (14 ± 2.5 versus 2.5 ± 1; *p* = 0.049). Together with significant pathways of 'activation of metabolism' and 'protein metabolism', this observation indicates increased turnover in tendon constructs derived from older MSC donors. A summary of the number of neopeptides identified in each condition is presented in Table 5. Very few neopeptides were identified for non-collagenous proteins whilst a large number of collagen proteins demonstrated neopeptides.

**Table 5:** Number of neopeptides identified in each condition for a range of collagens and glycoproteins

	Mean number of neopeptides	
Protein	Young	Old
COL1A2	0.0	1.0
COL2A1	0.0	1.0
COL5A1	0.0	1.3
COL6A1	2.0	2.3
COL6A2	0.0	1.0
COL6A3	0.0	2.8
COL8A1	1.5	1.3
COL12A1	1.0	4.0
Thrombospondin 1	2.0	3.3
Tenascin	1.0	1.8
Total	5.0	14.0

## Discussion

MSCs are an appealing source for cell-based treatment of musculoskeletal diseases and injury. Ageing is associated with various altered cellular phenotypes. Furthermore, the regeneration potential of MSCs is reduced with increasing age and is correlated with changes in cellular functions [5, 24]. This study of chondrogenic, osteogenic and tenogenic constructs derived from young and old MSCs provides a comprehensive proteomic analysis of tissue constructs whilst concurrently enabling improved understanding of the age-related functional and biological variations, which may affect their applications to regenerative medicine. In addition, our approach enables common and tissue-specific pathways of musculoskeletal ageing in an in-vitro system to be identified.

Characterisation of the tissue constructs was undertaken using standard methods following chondrogenic and osteogenic differentiation. For tenogenic constructs we used histological staining with Masson’s Trichrome and TEM to ascertain the presence and organisation of a collagenous matrix together with *collagen type I*, *THBS4* [40] and *SERPINEF1 *gene expression. The latter two have recently been identified as being the most DE genes in tendon differentiation but with low expression in chondrogenic differentiation [38]. There was heterogeneity in the response to differentiation of these markers which was not age related, similar to that in aggrecan expression in chondrogenic constructs. The conditions of differentiation may impact on results. Because differentiation involves culture with different factors, which change according to the method used, it is conceivable that there might an age-related change in response to alteration of these factors which could affect results. Further work is required in this area.

The proteomic profiles of the constructs demonstrated using 1D gels showed that there was no gross difference in the profiles within construct type with ageing. When these results were compared with the number of identified proteins there were a higher number of protein identifications within the osteogenic (2233 proteins) and chondrogenic (2226 proteins) constructs, indicating a more complex proteome in these tissues. This was similar to the number of proteins identified in MSCs (2347 proteins) [24]. A Rapigest™-based protein extraction workflow was utilised for tenogenic (615 proteins) constructs whereas a guanidine-based extraction was used for chondrogenic and osteogenic constructs, because Rapigest™ provided superior results compared with guanidine for tenogenic constructs in terms of proteins identified. The difference in number of protein identifications was probably due to the altered cell to ECM ratios within constructs, evident from histological sections. One limitation of the study is that it cannot be ruled out that some of the proteome pathway differences evident could be due to the culture format or duration.

We used label-free quantification to identify age-related DE proteins within each construct type. For our initial analysis we filtered data using *p* and *q* values (false-discovery adjusted *p* values for multiple testing) in order to reduce the number of false positives [41]. This produced 128 DE proteins in chondrogenic constructs, 207 DE proteins in tenogenic constructs but only four proteins for osteogenic constructs. This indicates that at this level of filtering the age of the MSC donor has little effect on the proteome of osteogenic 2D constructs, whilst the protein composition of tendon constructs is most affected by MSC donor age. This disparity with the other two construct types could be due to their differentiation in 3D. We used standard 3D construct differentiation techniques for chondrogenic constructs due to problems with dedifferentiation into monolayers [42]. The pellet characteristics closely mimic cartilage [43]. In tenogenic differentiation, uniaxial tension in 3D is a requirement for differentiation [44]. For osteogenic constructs we used the standard 2D system because it is the best described method and we could foresee problems with the mass spectrometry compatibility of materials included in many of the 3D osteogenic systems [45]. However, 2D techniques inadequately produce the in-vivo environment for stem cells established by extrinsic and intrinsic cell signalling affecting biological function and differentiation capacity over time [46]. Therefore, the effect of MSC donor age on the 3D osteogenic construct proteome should be studied in future.

For the musculoskeletal constructs it is essential to demonstrate the cellular phenotype and tissue composition, especially the ECM molecules that play a structural role and that contribute to the resulting mechanical properties. Therefore we identified the compositional and age-related DE matrisomal proteins in constructs. A number of matrisomal proteins were shared between all constructs such as COMP, TIMP1, decorin and biglycan, whilst some were shared between some types such as TIMP3 between chondrogenic and tenogenic constructs. This demonstrates that, similar to native tissues, the constructs have contrasting ECM profiles [20, 47]. Furthermore, when DE matrisomal proteins were investigated some proteins again shared age-related changes (COL4A2, MXRA5, THBS1 and MMP14) in chondrogenic and tenogenic constructs. Others such as plasminogen in chondrogenic constructs, and lumican in tenogenic constructs, were construct distinct. Interestingly, in agreement with results from 1D gels, the tenogenic constructs contained the most ECM matrisomal proteins as a percentage of all proteins identified within the construct type. Our findings revealed that the age of the donor MSCs had distinct or similar effects on construct ECM, depending on the differentiation lineage. The consequence of these altered matrices on the mechanical competence of the constructs requires further work.

Gene ontology revealed that metabolic processes were overrepresented in tenogenic constructs. This was also evident using IPA, which identified an increase in glucose and protein metabolism (both identified as activated in ageing), the latter related specifically to protein expression, proteolysis, catabolism and anabolism. Protein metabolism was demonstrated, for example, by an increase in abundance in old tendon constructs of TIMP-1, TIMP-3, MMP-2 and MMP-14. Furthermore, this was validated by neopeptide analysis, an indicator of protein turnover [20, 30]. Neopeptides represent ECM fragments produced by tissue remodelling. We have previously shown that neopeptide expression is altered in normal tendon ageing and disease [20]. The changes could be attributed to altered metabolism within the cells present in ageing tendon constructs. However, the increased abundance of proteases could also be due to release of intracellular proteases because of cell death. The DNA content of old tendon constructs was reduced despite all constructs being seeded at an equivalent rate at the start of the experiments. This could be due to cell death in older constructs or reduced proliferation capacity. However, because there was a concomitant increase in DE ECM proteins, these protein metabolism changes seem to be due to a dysregulation of protein metabolism in ageing tendon constructs. These findings may also help understand how the tendon undergoes physiological remodelling that is evident in ageing.

Gene ontology also identified that DE age-related proteins in chondrogenic constructs were higher for the cellular component ECM and extracellular region proteins compared with the other construct types, indicating that donor age affects matrix proteins of chondrogenic constructs the most. These age-related changes in the ECM could have important implications for the quality of engineered tissue.

There were protein changes in lipid metabolism-related proteins in chondrogenic constructs. In cartilage, lipids are a source of energy and are incorporated into structural components and signalling molecules. Chondrocytes express several proteins for fatty acid metabolism and cholesterol biosynthesis and these molecules are increased during chondrogenesis [48]. Chondrocyte lipid peroxidation has been suggested to have a role in cartilage ageing [49]. We identified age-related changes in proteins involved with LXR activation and cholesterol biosynthesis. Given previous findings, it would seem that MSC donor age has an impact on lipid metabolism which could affect their chondrogenic potential further given that our culture conditions were hypoxic.

A further interesting feature derived from pathway analysis was the demonstration of an age-related inflammatory response in chondrogenic constructs, a significant feature in chondrogenic constructs. Ageing MSCs are known to undergo inflammageing and tissue-engineered cartilage may be more predisposed compared with other tissue types, similar to native ageing cartilage [50]. Because MSC-derived chondrocytes are a potential treatment for chondral lesions [11], the use of allogeneic MSCs from younger donors may be beneficial in treating older patients. These findings demonstrate that our use of young and old donor-derived MSCs to produce musculoskeletal constructs could be a useful model to study musculoskeletal ageing.

A principal age-related feature of osteogenic constructs was mitochondrial dysfunction, when reactive oxygen species (ROS)-mediated oxygen stress overpowers the antioxidant defence system. Oxidative damage affects replication and transcription of mitochondrial DNA, leading to a decline in mitochondrial function and enhanced ROS production with further damage to mitochondrial DNA. In all constructs we demonstrated age-related protein changes involved in cell death and survival, and the alterations in oxidative stress probably contribute to this. Our results imply that MSC-derived tissue engineering from older donors must focus on oxidative stress protection.

Pathway analysis revealed that actin cytoskeleton changes were common to all ageing constructs. Others have identified an age-affected alteration in cytoskeletal organisation in rat MSCs [51]. In all ageing constructs, similar to MSCs [24] we hypothesise that there is a decline in responsiveness to mechanical and biological signals due to a less dynamic cytoskeleton. The age-related network of proteins involved in cell migration and movement were also affected in all construct types. In total these findings are consistent with altered cytoskeletal dynamics affecting cell movement through coupling to actin organisation and turnover [52].

A number of upstream regulators of DE proteins were identified in each construct type. Our analysis revealed a number of significant transcriptional regulators potentially responsible for the protein changes. These data provide a starting point for future studies in MSC-derived tissue engineering of musculoskeletal constructs from older patients. One interesting finding was the contrasting roles of TGFβ and HIF1α in the DE proteins from chondrogenic and tenogenic constructs. TGFβ was significantly predicted to affect protein changes relating to tissue development in chondrogenic and tenogenic constructs and relating to differentiation in chondrogenic constructs, but in opposite directions; inhibited in chondrogenic but activated in tenogenic. Whilst there is only indirect evidence for a role of TGFβ in ageing, it is an important growth factor in development and differentiation. In our study, TGFβ signalling was predicted to be inhibited in chondrogenic constructs and activated in tenogenic constructs. This could be due to differing responses to oxidative stress previously identified here or distinctive requirements for culture conditions dependent on construct type. Furthermore, expression of the sets of age and tissue-specific transcriptional regulators may explain these findings, leading to similar molecular scenarios for some pathways (antioxidant, cell survival and cytoskeleton) but contrasting in others (e.g. protein and energy metabolism in tenogenic constructs and lipid metabolism in chondrogenic constructs).

Finally, the functions of the DE proteins identified which relate to cell death and survival, and antioxidant and cytoskeletal changes, are associated and important for chondrogenic, osteogenic and tenogenic differentiation. Chondrogenesis is characterised by changes in cell shape [42] and actin organisation is essential [53]. In tenogenesis, cytoskeletal organisation is also paramount [54]. Furthermore, osteogenesis is tightly regulated by ROS (reviewed in [55]). Age-related proteomic changes will thus affect the ability and quality of tissue-engineered constructs.

The study of musculoskeletal ageing in bone, cartilage and tendon is generally undertaken in isolation and it is often difficult to attain aged matched tissue samples in humans. We propose our approach as a model for musculoskeletal ageing that could be probed further to identify factors that may aid in recapitulation of a younger tissue phenotype. This is because as musculoskeletal tissues age they become more prone to age-related musculoskeletal disease such as osteoarthritis, tendinopathy and osteoporosis. We have identified some shared age-related characteristics (inflammageing, oxidative stress, cytoskeletal) which raise the prospects that common therapeutic targets could be developed to prevent these diseases. Understanding what drives these changes in diverse tissues could lead to the development of new therapeutic methods, which are advantageous to the musculoskeletal system in general.

## Conclusions

Mass spectrometry-based proteomics provides an efficient method to monitor the complete profile of cellular and ECM molecules from tissue-engineered constructs. The differentially regulated proteome characterised by this study can potentially guide translational research specifically aimed at effective clinical interventions. Our data have provided valuable clues for our better understanding of the underlying mechanisms that are responsible for age-related changes in tissue-engineered constructs, thus assisting in the application of MSCs in cell-based therapy for cartilage, bone and tendon regeneration. These results also have significant implications for therapeutic cell source decisions (autologous or allogeneic), revealing the necessity of approaches to improve functionality of ageing MSCs.

## Additional files

Additional file 1: Table S1. Presenting the primer sequences. (XLSX 11 kb) Additional file 2: Table S2. Presenting details of the antibodies used for validation studies. (XLSX 10 kb) Additional file 3: Table S3. Presenting the proteins identified in PEAKS PTM using the human reviewed database. (XLSX 425 kb) Additional file 4: Figure S1. Showing a Venn diagram of the ECM proteins identified in constructs using MatrisomeDB. (DOCX 100 kb) Additional file 5: Table S4. Presenting the classification of proteins in chondrogenic, osteogenic and tenogenic constructs using MatrisomeDB. (XLSX 23 kb) Additional file 6: Table S5. Presenting results of the DE proteins identified in constructs made from young and old MSCs using label-free quantification with ProgenesisQI™. (XLSX 52 kb) Additional file 7: Table S6. Complete list of significantly expressed proteins and DAVID analysis of the expression patterns. (XLSX 297 kb) Additional file 8: Table S7. Presenting upstream log ratio molecule predicted activation *p* values of target molecules in the dataset. (XLSX 25 kb)

## Abbreviations

BM-MSC: Bone-marrow-derived mesenchymal stem cell
COL: Collagen
COMP: Cartilage oligomeric matrix protein
DAVID: Database for Annotation Visualisation and Integrated Discovery
DE: Differentially expressed
ECM: Extracellular matrix
FDR: False discovery rate adjusted
GAPDH: Glutaraldehyde-3-phosphate dehydrogenase
HIF1α: hypoxia-induced factor-1 alpha
IPA: Ingenuity pathway analysis
LC-MS/MS: Liquid chromatography tandem mass spectrometry
MMP: Matrix metalloproteinase
MSC: Mesenchymal stem cell
MXRA: Matrix remodelling associated
PANTHER: Protein ANalysis THrough Evolutionary
PRELP: Proline/arginine-rich end leucine-rich repeat protein
qRT-PCR: Qualitative real-time PCR
RUNX2: Runt-related transcription factor 2
SDS-PAGE: Sodium dodecyl sulphate polyacrylamide gel electrophoresis
SERPINE: Serpin peptidase inhibitor
SOD: Superoxide dismutase
SOX9: SRY Box9
TEM: Transmission electron microscopy
TGFβ: Transforming growth factor beta
THBS: Thrombospondin
WISP2: WNT inducible signalling pathway protein 2
