# Supplementary material for: A proteomic analysis of chondrogenic, osteogenic and tenogenic constructs from ageing mesenchymal stem cells
Source: Stem Cell Res Ther. 2016 Sep 14;7(1):133. doi: 10.1186/s13287-016-0384-2 (PMC5022190; doi:10.1186/s13287-016-0384-2)

Supplementary Information Figure 1- Venn diagram of the extracellular matrix proteins identified in constructs using MatrisomeDB. Proteins were identified with the Unihuman reviewed database, with at least 2 unique peptides, a 2-fold change in expression and q<0.05. These proteins were input into MatrisomeDB. A list of the proteins within each construct type and those shared is in supplementary file 4.


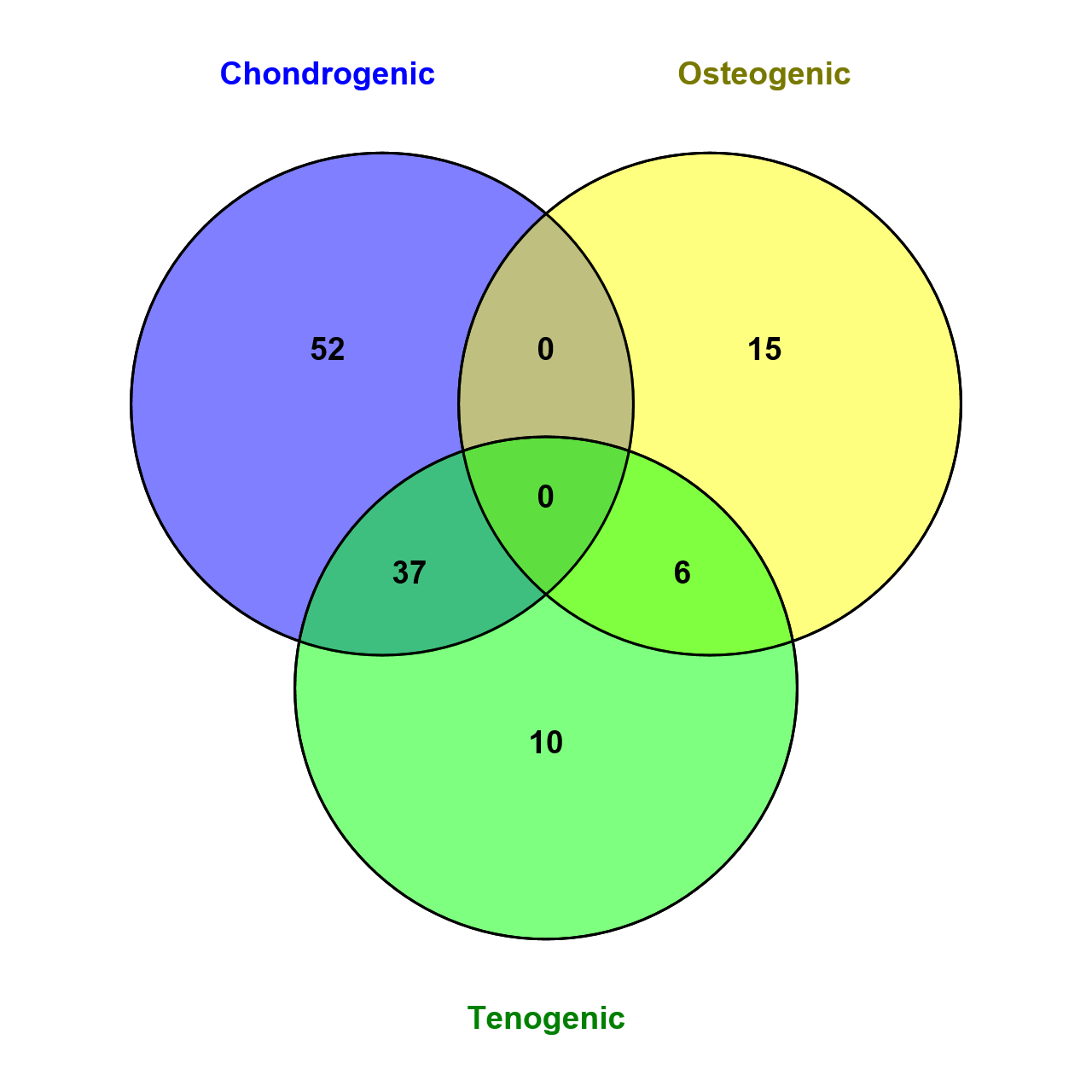

Supplement: Additional file 4: Figure S1. — Showing a Venn diagram of the ECM proteins identified in constructs using MatrisomeDB. (DOCX 100 kb) [file 13287_2016_384_MOESM4_ESM.docx]
